# American Academy of Dental Science

**Published:** 1893-09

**Authors:** 

**Affiliations:** American Academy of Dental Science


### Reports of Society Meetings.

### AMERICAN ACADEMY OF DENTAL SCIENCE.

The regular monthly meeting of the American Academy of Dental Science was held in the Boston Medical Library Association rooms on April 5, 1893, at 7.30 P.M., President Brackett in the chair.

*President Brackett.*—Our first subject for discussion is, "To what extent and under what conditions is the collar-crown a cause of pericemental inflammation?"

*Dr. Allen.*—The collar-crown, when properly fitted, is, in my opinion, rarely, if ever, a cause of pericemental inflammation.

When inflammation is present, it is usually due to one of three causes. And these are a lack of coaptation between the collar and the root, the presence of particles of cement or fragments of tooth under the gum after the crown is set, and a rough or unfinished edge in the collar.

There is probably no operation in dentistry which, if skilfully performed, yields more satisfaction than the setting of a collar-crown, nor one which, if performed in an unskilful or slovenly manner, is more thoroughly unsatisfactory.

*Dr. Fillebrown.*—In my opinion, collar-crowns are not likely to produce pericemental inflammation if properly adjusted. The troubles which arise are from mal-adjustment; the crown being too large or the root not properly dressed down, or the band going up too far under the gum at some one point. I have set a great many of them, and expect to set many more. This crown, as a rule, has been entirely satisfactory and as little liable to produce inflammation as any kind of crown that has ever been used.

*Dr. Gillet.*—When I read the announcement of our subject on the card it seemed to me that there was not much to be said, except that, if the collar was properly fitted and well set, there would be trouble only in very exceptional cases.

In the course of conversation with a student the other day, he made a statement that helps explain why, in some instances, the collar-crown is a source of inflammation. Before coming to the dental school he had studied with a preceptor. During our conversation he asked if I approved of gold caps. I told him I certainly did approve of the cap in its proper place. He said he had no faith in a gold cap set as he had been taught to set it. On questioning him, I found that, according to his method, the roots were not trimmed at all; the cap was selected large enough to go easily into place; slits were cut in the edge, and when the cap was set an attempt was made to burnish it so as to fit at the edge. I suggested to him that the fault was with the operator rather than with the method of crowning.

*Dr. Stevens.*—I agree with others who have spoken that pericemental inflammation from collar-crowns is due almost altogether to ill-fitting bands. Many of those used are bands with level edges, there being no attempt at fitting them over the alveolar ridge; and they are struck on the top and forced into place. I have several times, when in the dental depots, seen models for which gold crowns were being selected by the dental clerks. The roots in the models had not been trimmed, and the object seemed to be to select crowns large enough to go over the roots, and then get the crowns into place as best they could. I presume the majority of the gold crowns that are put on are put on in that way.

*President Brackett.*—If there is nothing further to be said, we will proceed to the discussion of the second topic, which is, "The relative advantages of local and general treatment for obtunding sensitive dentine. Should arsenic ever be used for this purpose?"

This is certainly a practical matter in which no one of us, so far as I have knowledge, has attained the results he would like to attain, and in which general testimony and suggestion should be very profitable.

If we could control, modify, and annihilate the sensitiveness of dentine, we should rob our operations of a large part of their terrors; but in this field there is much yet to be accomplished. I hope the testimony of the evening will help on such a worthy, practical cause. I think we might do worse than to ask each gentleman in turn what he does in practice in the treatment of sensitive dentine. Shall we hear from you, Dr. Allen?

*Dr. Allen.*—I have no specific for this. I once thought I had, when the steam arrangement came upon the field. I found it to be useful, but somewhat disappointing in cases where I was very anxious to have things go off successfully. The pain that it caused has been the chief objection to its use. I now mainly depend upon subduing the nervous apprehension of my patient by careful preliminary treatment. This consists in being as calm and as reassuring as possible myself in order to relieve that dread which accompanies dental operations. I have also great faith in sharp instruments. When these means fail, and it is difficult to excavate, I simply go very slowly in burring out a cavity, using a sharp instrument. If it is so painful that my patient is inclined to stop the operation, I try to be still more gentle, and I say that as soon as it hurts it will be the signal for me to stop. That, however, is hardly apropos to the subject.

For an obtundent in severe cases I have used within three or four months the chloride of ethyl, which I consider is very valuable. In many cases it has been a specific. As for general, systemic treatment, I apply none, because I do not feel that it is within my province to prescribe drugs of doubtful action to subdue a condition which is going to exist three days later. I simply use means which the operation demands at the time.

*Dr. Williams.*—I don't know that it is worth while to repeat what I have already written in regard to preparatory treatment for endangered pulps and for sensitive dentine. Although I believe there are cases where no harm or after-suffering results from the excavation of a tooth in which the dentine is sensitive, yet there are cases where hard fillings are prematurely introduced which result in neuralgia or even abscesses. For such cases this preparatory treatment which I have referred to is the most satisfactory of any method of which I have knowledge. I shall not take your time to explain it here, as anybody who cares to look it up can find it in the INTERNATIONAL DENTAL JOURNAL some time within the last two or three years.

*Dr. Preston.*—I have never used many of these fancy things that are now advocated. If I come across a tooth that is very sensitive, I use chloride of zinc. It will generally reduce the sensibility, and so will phosphoric acid. But when the pulp is exposed I believe the only way is to destroy it, and I generally do it with arsenic. I used to put it in twice, but now I only put it in once, although in some cases repeated applications are necessary. Some operators used to cap the exposed pulp, but such pulps never amount to anything, no matter what they are capped with.

I brought this card to show you some of the pulps that I have taken out. Those light ones were taken out with an instrument when they were alive by introducing a piece of small barbed wire, giving it a little twist, and withdrawing it. The others were destroyed by putting in arsenic. This playing with the bare nerve does not answer. They had a system called the Huelian system a few years ago; I suppose you have all heard of it. Just above the edge of the gum you drill through and stab the pulp, which lets out the arterial blood and destroys the sensibility of the tooth; but it becomes a dead tooth. I never believed much in setting crowns on dead teeth.

A gentleman met me one morning at my door and said, "I want to ask you one thing, Did you ever extract a tooth and fill it and put it back again?" I replied, "Yes, I did that more than twenty years ago." He said that Dr. —— said he was the first one that ever did it.

I was looking up two or three of my cases to get at the dates, and the first one I found recorded was a Mr. Dix, dated August 29, 1840. Another one was a Mr. Russell, and the date was October 21, 1840. I met the latter gentleman about a year and a half after the operation; he stopped me and said, "If anybody wants to know about that tooth that you put back, tell them it is a first-rate one." Another case was that of a lady by the name of Harrington.

*Dr. Grant.*—I have no special lines to follow in obtunding sensitive dentine except to use very sharp excavators. In the way of drugs, the one thing that I have come to rely upon more than anything else is carbolic acid crystals. When there is a very sensitive tooth, I apply the rubber dam, take a carbolic acid crystal on an instrument, and let the acid drop into the cavity. I have found more people satisfied with that than with anything else that I have ever tried. It is not always successful, but, as a general thing, patients say that it does obtund the sensibility. In this way I have excavated teeth when I hadn't the least faith that I could control the sensitiveness, having made the application more to ease the mind of the patient than anything else. I believe in sharp excavators above everything else, and the system by which I have been able to get excavators sharper than by any other, is the McLean system.

After McLean discontinued making disks I thought I would try the black rubber corundum disks that I had been using for separating. I tried sharpening an excavator with one of these, and was surprised to find the keen edge that it gave. You can get an edge that is perfectly true, and with great rapidity. On an oilstone it is about impossible to secure a true edge, but with this disk you can make it exactly as you want it.

*Dr. Andrews.*—I have tried almost everything which has ever been suggested. Our patients demand something. I have used with some degree of success a mixture of chloroform, menthol, and cocaine. I do not remember now the proportions, but I think Dr. Eames has spoken before the society of this same preparation. But the best obtunder is a sharp instrument, and I wish here to enter a word of praise for the mechanically perfect burrs of the S. S. White Dental Manufacturing Company. I have used the Smalls instrument with some degree of success, but there were so many cases where it did not work satisfactorily that I gave it up. Hypnotism I have never tried. The method advocated by Dr. Williams has a good deal of merit. I thoroughly believe in preparatory treatment.

Some years ago Dr. Chandler spoke of finding the bicarbonate of soda tablets, such as are put up by Wyeth, of Philadelphia, an excellent thing to use in very sensitive cases. One or two of the tablets should be given at night for a few days before an appointment. I have tried this method in several cases and believe it to be an excellent thing.

*Dr. Cutter.*—My plan is to get the cavity as dry as possible, and to use sharp instruments. I use the McLean process for sharpening.

*Dr. Payne.*—I use a warm saturated solution of cocaine in alcohol with very good success, and in the very sensitive cavities that I cannot obtund with that I use a preparatory filling of oxychloride of zinc. This filling is allowed to remain three or four months unless the pulp would be thereby endangered.

*Dr. Gillett.*—I have tried almost everything that I could hear of or could find. Until lately I have relied principally on Dr. Waitt's obtundent. I don't know whether he has any special claim to it, but it is put up and is for sale under his name. Its chief ingredient I understand to be caustic potash, and the result is obtained by the process of desiccation. It is sometimes very painful. Some patients like to have me use the steady current of slightly-warmed air, which dries the cavity very thoroughly and makes it less sensitive, sometimes completely removing sensation.

*Dr. Stevens.*—I find nothing better than dryness and a sharp instrument. The cavity should be allowed plenty of time to dry. In case a tooth is extra sensitive it is a good plan to apply the rubber dam to obtain dryness and leave the tooth for a while, doing work upon a less sensitive one. When the sensitive tooth is returned to, there will be much less pain and perhaps none at all. There is a great deal in handling the patient. I suppose Dr. Fillebrown would call it "hypnotism." We have many patients who nerve themselves up to the operation to such an extent that they will jump if we do not hurt them at all. I try to get such patients to let go of themselves. I don't know of any better way of expressing it. I have had patients who, after learning how to relax themselves, were able to have their teeth worked on with comfort and with no fear or dread of the operation.

*Dr. Clements.*—I rely mainly on dryness and sharp instruments, but in addition I use a remedy which is called "Robinson's Remedy." It contains carbolate of potash and possibly some glycerin. When applied warm I rarely find any objection to it, and it certainly will obtund all sensitiveness. But I think with sharp instruments and skilful handling nearly everything that is necessary can be done without the use of an obtundent, except in cases of teeth which are hypersensitive or where there is a great deal of trepidation. Of course no gentleman here would ever think of using a very large ligature of twine and tying it tightly around the teeth, thereby causing a great deal of pain and making the tooth extremely sensitive.

*Dr. Smith.*—I invariably use the rubber dam, and consequently all cavities that I excavate, or a large percentage of them, are dry. And yet dryness does not fully remove sensitiveness. I have used many different obtundents; the one which I have used considerably of late is a warm solution of cocaine and alcohol. I have also been using an obtundent advertised by B. L. Knapp & Co., which was first called to my attention by Dr. Eddy. He had used it with considerable success; it seems to accomplish more than the solution of cocaine and alcohol. To-day I treated a cavity on the buccal surface of an inferior wisdom-tooth; half of the cavity going down under the margin of the gum. The clamp was applied with the rubber dam and the tooth was made perfectly dry.

I would say right here that I always test a cavity to see whether it is sensitive or not; there are many cavities not sensitive without the use of obtundents. In the case that I speak of the cavity was thoroughly dried by alcohol, then dried again by means of warm air from a chip-blower. I then took a sharp instrument and excavated carefully, with the result of producing excruciating pain. I made an application of the Knapp obtundent,—I don't know whether that is the name of it, but that is the way I have it on my book,—and I waited twelve minutes by my watch. At the end of that time I was able to excavate without pain. And here let me say that we often do not wait long enough before commencing an operation. If you have several cavities under the rubber dam at one time you can work on the one which is the least sensitive first; but if you have only one cavity you must wait. I suppose my professional brother here would say that I hypnotize the patient. I tell them that this remedy is always sure, and say to them, "Now I am going to wait twelve minutes, and at the end of that time I shall commence to excavate and you will not feel it at all." And usually they do not. Whether it is because they believe what I tell them or because of the virtue of the obtundent, I cannot say; I think it is the obtundent. In some cases this obtundent has not worked perfectly, but it always obtunds in a degree; and possibly in the less satisfactory cases, if I had made a second application and waited, the result would have been very different.

*Dr. Fillebrown.*—I have found dryness an excellent thing, and of the drugs I have found cocaine the most successful. But the most desirable and successful of all is the application of oxyphosphate of zinc. By allowing this to remain for a time we get an improvement in the general condition of the tooth by the deposition of lime salts, and it is better able to stand the shock of excavating.

I know you are all prepared to hear me speak of the use of hypnotic suggestion in this connection. I shall not disappoint you. It has certainly been the most powerful and generally successful and satisfactory of any means that I have ever used, and it has stayed by me longer than anything I have ever tried. You know we often take up some new thing and use it in a number of cases, and it fails so often that we soon get discouraged and try something else, and so keep changing. But for a whole year this has continued giving great satisfaction and comfort to both my patients and myself, and I think more of it now and have better results with it to-day than ever before. I will say just this to Dr. Stevens, that if instead of asking his patients (to use his own phraseology) "to let themselves down," he will try active suggestion, making it positive, and saying, "Now this does not hurt you," "Your muscles are relaxing," "Your tooth is becoming anæsthetized," "You are resting," making it obligatory on the patients to receive suggestions, a great deal more can be accomplished. I am speaking now not in reference to a patient who is hypnotized, but one that is not. I had a boy in my chair this afternoon, twelve or fifteen years old, whose teeth have always been sensitive. I said to him, "Let me hypnotize you;" but he said, "Oh, no, no!" so I commenced talking to him in the manner which I have described, and the little fellow settled right down and bore the operation,—not without some pain, but with comparative comfort. The only difference between the patient who is hypnotized and the one that is not is this: if a person is hypnotized he will listen more readily to the suggestions and you will be able to make them more effective. In the opening remarks of Dr. Eames, he says, "Sensitiveness of the teeth depends upon a condition of the nerve-centres, and these centres are what we want to control. Now, it has been established over and over again by myriads of circumstances that the nerve-centres can be affected by audible suggestion so that they will fail to respond to sensations produced by a shock to the teeth or any other part. This is a physiological condition, and we may as well accept it as a fact and use it. Those of you who have not considered this before, I ask to try the active suggestion; you are not dealing with an uncanny subject; you will find it produces a wonderful effect on your patients and will prove a most effective obtundent for sensitive dentine.

We all know that fear is our greatest enemy, and if we can remove fear from our patients we can get along very well. I had a patient to-day who came four hundred miles simply because she thought I could relieve her of the terrible dread she had of dental operations, and make it possible for her to have some necessary work done. It seems that when she was a young woman a dentist thrust an instrument into an exposed pulp and sought to remove it by immediate operation. In describing her sensations, she said, "For several moments I thought I was dead." Since that time she has been utterly unable to sit down in a dentist's chair. Seeing that she was willing to be hypnotized, I tried it last Friday for the first time and with entire success. She has been to my office four times since then, and sat entirely quiet for a good many hours and had no trouble at all. To-day I wanted to examine a dead tooth and I did not think it needed any suggestion, but she stopped me and said, "No, the terror makes it simply impossible for me to bear it." I suggested the removal of the terror, and there was not a quiver from her afterwards.

*Dr. Williams.*—Dr. Fillebrown's remark that if you tell patients that you will not hurt them, you really do not, reminds me of a story I came across the other day of a boy whose mother had full faith in this sort of thing under another form. The boy had the toothache and his mother took him to a mindcurer, who went through some motions with his hands and said, "Your tooth does not ache now." The boy looked up at him and said, "You lie, it does." Now, sometimes it happens that they will not believe you when you tell them that you are not hurting them.

A good many years ago a friend of mine suggested that we go and take some lessons on this subject,—biology, as it was then called. We took a course, and I practised on several patients, and one day a gentleman asked me if I thought I could fill some teeth for his wife, a lady of very nervous temperament who could not bear to have anything done by the dentist. She came to my office and the experiment was entirely successful, as she underwent a very severe operation without the slightest pain. I remember there was a large cavity in a lower first molar in which the pulp was exposed, and I cut into the pulp during this hypnotic state and took it out and cleared out the canals and dressed the roots. That I consider about as sharp a test as could be made with any anæsthetic. So there is something in it. I found, however, that it took up much time.

*Dr. Fillebrown.*—I don't believe that hypnotic suggestion or anything else will obtund in every case or often sufficiently to extract a pulp.

*Dr. Williams.*—My case was certainly one in which it sufficed.

*Dr. Fillebrown.*—There are limits to all things human, and I would not have any one think for a moment that anything that I

use is with me absolutely successful in all cases, or more than seldom in very severe cases.

*Dr. Stevens.*—Dr. Fillebrown is certainly progressing, judging from his remarks to-night, and I think he will find as time goes on that he will not need to use the verbal suggestion; he can use mental suggestion with very good effect. Some people hold that a person cannot be hypnotized without giving consent. I have seen something of hypnotism for many years, and I know from practical experience that such is not the case.

I don't know whether any of the gentlemen present have used what is called "Thayer's Obtundent," either for sensitive dentine or for application to the gums. I have found it very nice, especially for application to the gums in applying ligatures. I applied it to open an abscess for my sister, and found afterwards that in this case the skin turned white and sloughed off. Dr. Draper used some of it, and it produced the same result. There is no complaint from the patients and no bad after-results; but the appearance is unsightly.

*Dr. Williams.*—I should say the moral of that was not to use anything the composition of which you do not know.

*Dr. Fillebrown.*—My own experience is not sufficient to settle the question whether it is possible to hypnotize without consent of patient; but the literature on the subject states that only in very exceptional subjects would it be possible to hypnotize without passive consent and utterly impossible with active resistance.

*Dr. Williams.*—From my observations, that would depend somewhat on how much a person had been accustomed to being hypnotized. Sunderland, the famous lecturer, used to go through the audience and fix his eyes on some one and mentally will that person to get up and go on the stage. In that way he picked out his most impressible subjects and amused the rest of the audience.

*Dr. Andrews.*—That matter recalls an incident which took place in my uncle's office years ago. This same Dr. Sunderland was present when a teamster came in to have several teeth extracted. He went up to the man, made a few passes, and told him to sit down in the chair, and several difficult teeth were then extracted without his knowing it. Dr. Sunderland slapped the man on the back, and, of course, he came out all right and went away feeling happy. That was done without the man's consent or knowledge that he was going to be hypnotized.

*Dr. Stevens.*—I have seen the same thing done.

William H. Potter, D.M.D.,
*Editor American Academy of Dental Science.*

